# Ligands in PSI structures

**DOI:** 10.1107/S1744309110008092

**Published:** 2010-07-06

**Authors:** Abhinav Kumar, Hsiu-Ju Chiu, Herbert L. Axelrod, Andrew Morse, Marc-André Elsliger, Ian A. Wilson, Ashley Deacon

**Affiliations:** a Joint Center for Structural Genomics, http://www.jcsg.org, USA; b Stanford Synchrotron Radiation Lightsource, SLAC National Accelerator Laboratory, Menlo Park, CA, USA; c Center for Research in Biological Systems, University of California, San Diego, La Jolla, CA, USA; d Department of Molecular Biology, The Scripps Research Institute, La Jolla, CA, USA

**Keywords:** structural genomics, ligands, PSI, protein–ligand complexes, data mining

## Abstract

Approximately 65% of PSI structures report some type of ligand(s) that is bound in the crystal structure. Here, a description is given of how such ligands are handled and analyzed at the JCSG and a survey of the types, variety and frequency of ligands that are observed in the PSI structures is also compiled and analyzed, including illustrations of how these bound ligands have provided functional clues for annotation of proteins with little or no previous experimental characterization. Furthermore, a web server was developed as a tool to mine and analyze the PSI structures for bound ligands and other identifying features.

## Abstract 2

A survey of the types and  frequency of ligands that are bound to PSI structures is analyzed  as well as their utility in functional annotation of  previously uncharacterized proteins.

## 1. Introduction

International structural genomics initiatives, including the US-based Protein Structure Initiative (PSI; http://www.nigms.nih.gov/Initiatives/PSI/), have led to an unprecedented increase in the rate at which new protein structures are being solved and made available to the scientific community (Levitt, 2007 ▶). To date, these efforts have contributed over 7500 protein structures to the Protein Data Bank (PDB; Dutta *et al.*, 2009 ▶), more than half of which have come from the PSI. For the most part, the PSI effort has focused on determining unique structures from protein families that previously lacked any structural representative and on providing better structural coverage for large diverse protein families where more structures are needed to accurately model the entire family. Consequently, many of the proteins solved have little or no previous experimental characterization and have been classified as domains of unknown function (DUFs; Bateman *et al.*, 2010 ▶) or have only a tentative functional annotation based on amino-acid sequence homology. A variety of online tools and web-based search engines, such as *EBI-SSM* (Krissinel & Henrick, 2004 ▶), *DALI* (Holm *et al.*, 2008 ▶), *VAST* (Gibrat *et al.*, 1996 ▶) and *fast-SCOP* (Chi *et al.*, 2006 ▶), allow the inference of function based on structural similarity. However, these approaches have their limitations.

A significant number of the structures solved by structural genomics efforts can be assigned to superfolds (Orengo *et al.*, 1994 ▶), such as TIM-barrel and ferredoxin folds, whose members perform a wide diversity of biological functions. Thus, knowledge of the structure is often not sufficient to deduce the exact cellular function of a protein. To further aid in functional annotation, additional methods can be explored, such as catalytic residue matching and analysis of protein surface properties, although these methods usually only partially enhance the functional assignment (Binkowski *et al.*, 2005 ▶; Laskowski *et al.*, 2005*a* ▶,*b* ▶; Porter *et al.*, 2004 ▶).

Another challenge faced by large-scale structural biology efforts is to effectively disseminate the structural results to a broad scientific community. Although all of the PSI structures are deposited immediately into the PDB and rapidly released, only a small fraction of them have been described in publications in the scientific literature. Recently, efforts have been made to develop more streamlined web-based tools to rapidly disseminate key findings and new insights derived from these structures, as exemplified by the PSI Knowledgebase (http://kb.psi-structuralgenomics.org) and The Open Protein Structure Annotation Network (TOPSAN; http://www.topsan.org/; Krishna *et al.*, 2010 ▶). However, it is clear that complementary user-friendly tools would be extremely beneficial to mine the latest structural data for functional and methodological insights. A rich source of functional data that is often overlooked in the PSI structures are the ligands that are identified during interpretation of the electron-density maps and subsequently built into the deposited structures. More than half of the PSI structures (65%) contain bound ligands, such as metal ions, cofactors, substrates and effectors. Many of these ligands are acquired during protein production, whereas the remainder are incorporated into the protein at various steps during the purification and crystallization stages, as, for example, buffer reagents, salts, precipitants and cryoprotectants. In many cases, these non-native ligands act as surrogates for the natural ligands owing to their similar biophysical properties. Their identification can often pinpoint favorable electrostatic regions or ‘hot spots’ on the protein and these surrogates often mimic the natural ligand–protein interactions, thus providing functional clues and insights.

The Joint Center for Structural Genomics (JCSG; http://www.jcsg.org) has designed the Ligand Search Server to be a fast and intuitive way to mine the PSI structures for detailed information regarding bound ligands. Searches can also be readily generated for entire families or for distinct classes of proteins or ligands, thus furthering collation and analysis of the functional knowledge derived from otherwise diverse sets of structures.

## 2. Methods

### 2.1. The Ligand Search Server

**Figure 1:**
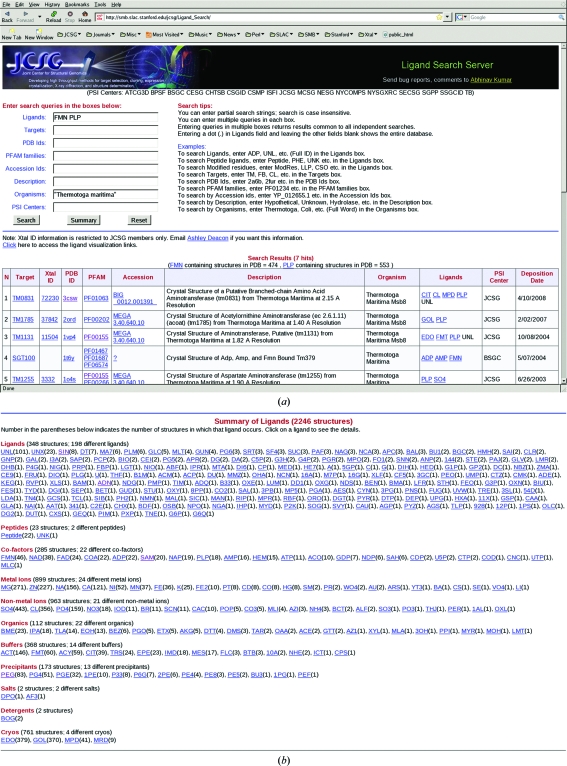
The Ligand Search Server and an example of its use. (*a*) The server’s main page showing the search form and search example looking for PSI structures that contain either FMN or PLP bound to proteins from *Thermotoga maritima*. Tips on how to use the interface are displayed on the right and a partial list of structures is listed at the bottom. (*b*) A summary of all of the ligands bound to the PSI structures is displayed when the ‘Summary’ button is clicked.

The JCSG Ligand Search Server (http://smb.slac.stanford.edu/jcsg/Ligand_Search/) was created to mine PSI structures and to identify and classify the different types of bound ligands whether of functional relevance or not. The server also serves as a portal to complementary sites such as the Protein Data Bank (PDB; http://www.pdb.org), TOPSAN and Pfam (http://pfam.sanger.ac.uk; Finn *et al.*, 2008 ▶) which facilitate further exploration. The main user interface provides eight different search fields, including (i) the PDB ligand code, (ii) the PSI target name, (iii) the PDB code, (iv) the Pfam accession, (v) the protein/gene product accession ID, (vi) the structure description, as listed in the title of the PDB header, (vii) the source organism name and (viii) the name of the PSI center. Each of these fields accepts multiple entries that are combined with a logical ‘or’ and entries in any of the eight search fields are then combined with a logical ‘and’ to generate the search query. A few search tips and examples are listed alongside the search form on the main page (see Fig. 1 ▶).

The ‘Search’ button submits the query against a locally maintained database which contains information on all of the PSI structures deposited in the PDB. The query results are returned as a single page that contains a concise tabular report at the top, which contains a row for every PDB structure that matches the query, lists the protein identifier used by the individual PSI center, the PDB code, the Pfam family name, the gene accession ID, the structure description, the source organism name, the bound ligands, the contributing PSI center and the deposition date. An additional column, ‘Xtal ID’, is included for JCSG structures which provides a link to specific information on the crystal used for structure solution, including all of the data and log files produced at various stages of structure solution and refinement. Most of the report fields are linked to other web resources to explore the structures further. This tabular report can also be exported to an Excel spreadsheet. Next, a ligand-visualization section provides links to HIC-Up (http://alpha2.bmc.uu.se/hicup/; Kleywegt, 2007 ▶) and Ligand Expo (http://ligand-depot.rcsb.org; Feng *et al.*, 2004 ▶) for each of the ligands found. Several summary sections that include information on the nature of the ligands found, the associated Pfam families and the source organisms follow. A ‘Summary’ button is also provided, which if used without any search query will generate an overall statistical report on all of the PSI structures in the database. More concise ‘Summary’ reports can be produced by including query values in the form fields.

### 2.2. Treatment of ligands at JCSG

During structure determination at the JCSG, attempts are made to account for all significant electron density observed during refinement. In addition to solvent molecules and chemical reagents used during protein production and crystallization, potential biological molecules, such as enzyme cofactors, substrates, products or their derivatives, which are presumably relevant to the protein’s function and clearly supported by the electron density and chemical environment, are modeled into the structures, even if these molecules were not explicitly present in the reagents used during the protein preparation and crystallization stages.

The JCSG routinely uses X-ray fluorescence to identify metals that are bound in the structures. This technique allows the identification of most metals in the sample with a single experimental spectrum. When multiple metals are detected, X-ray diffraction data sets are then collected above and below the relevant X-ray absorption edges of the metals and anomalous difference Fourier maps are calculated in order to unequivocally locate and confirm the identity of the bound metals. Lighter metals, such as Mg and Na, cannot be determined by X-ray fluorescence owing to limitations in our experimental setup; therefore, these are usually identified based on their binding geometries and environment.

Nevertheless, in many cases a suitable ligand cannot be unambiguously assigned to the electron density and the true identity of the ligand is inconclusive without further experimentation. The JCSG has adopted the policy of including these ligands as ‘unknown ligands’ and they are identified in the PDB file as UNL. The density is modeled by positioning a group of connected atoms that match the overall shape and a relevant description is included in the ‘REMARK 3’ field of the PDB header. To date, this strategy has surprisingly not been widely adopted by other PSI centers as it provides extremely valuable information that can be searched by a simple query; thus, the majority of these UNL-bound structures have been deposited by the JCSG (90%). Furthermore, all structures, including bound ligands, are internally peer-reviewed by at least one other scientist as a quality-control step prior to deposition in the PDB.

## 3. Overall statistics

A preliminary analysis of the 4200 currently available PSI structures shows that more than 2700 structures (∼65%) contain small-molecule ligands of some kind. These ligands can be loosely classified as biological ligands (substrate, products, cofactors, inhibitors and their analogs) or surrogates, as well as peptides, ions, buffer molecules, crystallization reagents and cryoprotectants. This classification scheme is described in more detail in Table 1 ▶ and Fig. 1 ▶. Most of the functionally relevant biological ligands, including cofactors, were not explicitly added to the crystallization experiments. Hence, these ligands are endogenous to the expression systems and were acquired during protein production.

**Figure 2:**
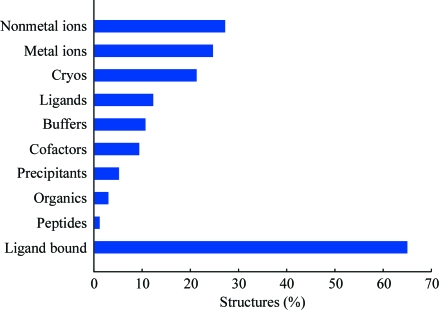
Percentage of PSI structures that have any small-molecule ligand bound to them. The small molecules are categorized by their types.

**Figure 3:**
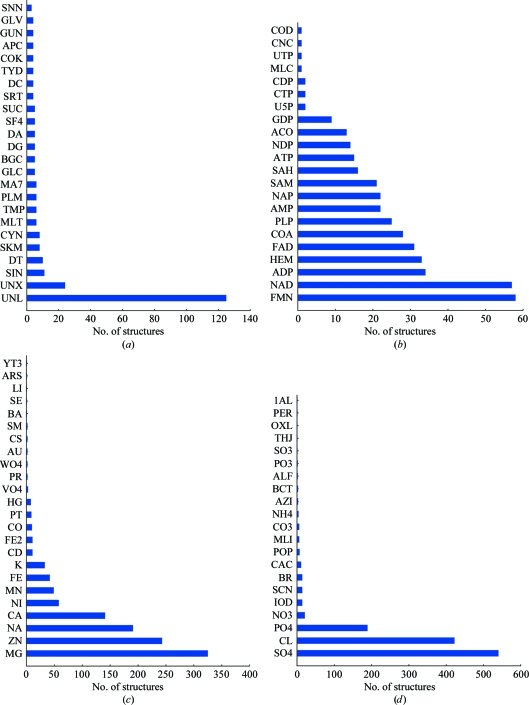
Distribution of various ligands by category and relative frequency. Only the most common of these small molecules are shown. The names of the ligands follow the IDs used in the PDB and their full names can be obtained from the Ligand Expo Server (http://ligand-depot.rcsb.org/ld-search.html). (*a*) The ‘Ligands’ category includes biological ligands, such as substrates/products or their analogs. (*b*) The ‘Cofactors’ category includes various cofactors of enzymes but excludes ions, which are shown in the ‘Metal ions’ (*c*) and ‘Non-metal ions’ (*d*) categories.

The overall distribution of the various types of ligands bound to PSI structures is shown in Fig. 2 ▶. It is of note that the JCSG reports more ligands in their structures compared with other PSI centers, particularly for the various ligands used as crystallization agents (buffers, precipitants and cryoprotectants); however, we also report more ligands in other categories. One possible explanation for this increased reporting of ligands comes from the standardized refinement and structure validation procedures implemented at the JCSG, in which specific steps (manual inspection and modeling of appropriate ligands) are undertaken to verify that all unmodeled electron density is properly accounted for. Indeed, a significant number of JCSG structures also contain ‘unknown ligands’ (UNLs), which refer to bound ligands that could not be unambiguously identified based on the electron density. The majority of these UNLs appear to be of biological importance since they are often located in crevices or cavities that resemble known active-site pockets or are identified based on comparison to structural homologs or other biochemical evidence. A survey of the number of biological ligands bound to PSI structures (Fig. 3 ▶) indicates that succinic acid (SIN), thymidine-5′-­monophosphate (DT) and palmitic acid (PLM) are the most frequently observed and are likely to originate from the expression system. Similarly, flavin mononucleotide (FMN), nicotinamide adenine dinucleotide (NAD) and flavin adenine dinucleotide (FAD) are the most common cofactors. Magnesium (Mg^2+^), zinc (Zn^2+^) and sodium (Na^+^) are the most common metal ions and sulfate (SO_4_^2−^), chloride (Cl^−^) and phosphate (PO_4_^3−^) are the most common non­metal ions that are found in PSI structures. These particular ions are often present in the expression, purification and crystallization solutions, which may account for their frequent observation. A further analysis of the biological ligands reveals that 25 are unique to PSI structures and have not been observed previously in other structures deposited in the PDB, again indicating the richness and diversity of the information that is being derived from such structure determinations of proteins of unknown function (Table 2 ▶).

## 4. Unknown ligands (UNLs)

**Figure 4:**
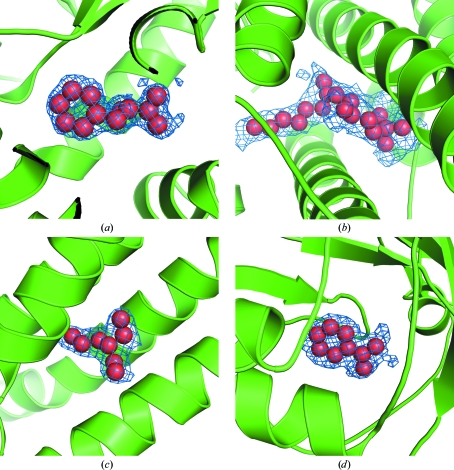
Unknown ligands (UNL) in a few PSI structures. The UNL atoms are represented as red spheres enveloped by electron-density mesh (2*F*_o_ − *F*_c_ density contoured at 1σ level above the mean) and surrounded by the protein rendered in cartoon representation. In many cases, the ligand could have been assigned as one or a few potential compounds, but is still annotated as a UNL since we have no definitive proof of its identity. (*a*) A protein of unknown function, NP_823353.1 from *Streptomyces avermitilis*, at 1.45 Å resolution. (*b*) A protein of unknown function possessing a ferritin-like fold (YP_832262.1; PDB code 3ez0) from *Arthrobacter* sp. Fb24 at 2.33 Å resolution. (*c*) A protein of unknown function from *Geobacter sulfurreducens* possessing a GGDEF domain (NP_951600.1; PDB code 3ezu) at 1.95 Å resolution. (*d*) Phzb2 (NP_250591.1; PDB code 3ff0) with a cystatin-like fold and an unknown function in phenazine biosynthesis from *Pseudomonas aeruginosa* at 1.90 Å resolution.

Examples of some UNL structures are shown in Fig. 4 ▶. About 75% of the UNL-bound structures now have some functional annotation and, therefore, biophysical and biochemical experiments can be designed to confirm the identity of the unknown ligands based on size and shape of the electron density as well as the nature of the environ­ment surrounding the bound ligand. For example, in several instances the UNL resembles benzoic acid or nitrobenzene (PDB codes 2f4p, 2ig6, 2pbl, 3d82, 3ecf, 3ejv and 3ff0). However, these compounds were not modeled as such since neither was present in any of the reagents used nor was there any correlation with the protein function. Uptake of endogenous molecules by proteins during the expression/purification stages is more common than is often appreciated, as exemplified by the occurrence of benzoic acid in 59 other structures in the PDB. However, in other cases, the UNL can provide functional clues about the protein. For instance, protein NP_823353.1 (PDB code 3giw) is annotated as a protein of unknown function (Pfam DUF574) with an unknown ligand bound (http://www.topsan.org/Proteins/JCSG/3giw). The UNL resembles phenyl­alanine and the protein is structurally similar to SAM-dependent methyltransferases (Martin & McMillan, 2002 ▶; Fig. 4 ▶ *a*), suggesting the possibility that it could be a phenylethanolamine *N*-methyltrans­ferase (PNMT; Wong *et al.*, 1992 ▶), histamine *N*-methyltransferase (HNMT; Rutherford *et al.*, 2008 ▶) or catechol-*O*-methyl transferase (COMT; Weinshilboum *et al.*, 1999 ▶).

## 5. Metals bound to PSI structures

Approximately 25% of PSI structures and 27% of the JCSG structures contain metal ions (Table 1 ▶). Zn^2+^ and Mg^2+^ ions are among the most prevalent ligands in PSI structures, with 5.7 and 7.6% occurrence, respectively. Of the 857 structures determined by the JCSG as of July 2009, 226 contained metal ions (50 Zn^2+^, 19 Fe^3+^, 28 Ni^2+^, 43 Mg^2+^, 41 Ca^2+^, 47 Na^+^, 11 K^+^, three Mn^2+^, two Co^2+^ and one Li^+^). The majority of Fe^3+^ and Zn^2+^ ions in PSI structures have a higher probability of being biologically relevant, since they are less frequently present in the crystallization buffers. For example, only 20% of the structures containing Zn^2+^ ions report a zinc salt in the crystallization conditions. Other metals are potentially less biologically relevant as they are more frequently used during protein purification or crystallization. PSI structures containing Ca^2+^, Mg^2+^ and Na^+^ ions were obtained when such salts were used in 77, 64 and 61% of their crystallization conditions, respectively.

The identification of a bound metal can often aid in identification of the active site in a protein. For example, the crystal structures of three proteins of unknown function, YP_164873.1 from *Silicibacter pomeroyi* DSS-3 (PDB code 3chv), YP_556190.1 from *Burkholderia xenovorans* LB400 (PDB code 3e49) and YP_555544.1 from *B. xenovorans* LB400 (PDB code 3e02), revealed structural similarity to 3-­keto-5-aminohexamoate cleavage protein (YP_293392.1) from *Ralstonia eutropha* Jmp123 (PDB code 3c6c), although their sequence identity (27–32%) is relatively low. Pairwise structural alignments gave a C^α^ r.m.s.d. of 1.6 Å for 264 aligned residues between 3chv and 3c6c, a C^α^ r.m.s.d. of 1.6 Å for 275 aligned residues between 3e49 and 3c6c and a C^α^ r.m.s.d. of 1.7 Å for 259 aligned residues between 3e02 and 3c6c. All four structures share a conserved Zn^2+^-binding site in which almost all of the active-site residues are identical. Other examples of using structural knowledge about a bound metal to enhance the functional annotation are presented elsewhere in this issue. Bakolitsa and coworkers provide an example of the identification of Zn and Ni bound to the structure of the DUF1470 protein (Bakolitsa *et al.*, 2010 ▶). Axelrod and coworkers provide another good example where binding of Zn^2+^ in the zinc-finger domain combined with structural comparisons suggest that two of the PF02663 Pfam family members in this study may bind nucleic acids and possibly function as transcriptional regulators (Axelrod *et al.*, 2010 ▶). These results have revealed functional and structural diversity within the PF02663 family.

## 6. Functional clues

### 6.1. Proteins of unknown function

Submitting the query ‘Unknown’, ‘Uncharacterized’, ‘Hypo­thetical’ or ‘DUF’ in the Description field of the Ligand Search Server finds 593 PSI structures (∼14% of the total) that lack any functional annotation. The vast majority (474 structures) have been assigned to families in Pfam based on their amino-acid sequence.

About 66% of these 600 or so functionally unannotated proteins have one or more bound ligands. A closer examination of those ligands that are most likely to be biologically relevant (excluding crystallization and cryogenic reagents, although in some cases these may also provide clues to function) indicates that the most frequently found are either metal ions (22% of all ligands) or ligands with unknown identity (UNL; 5%), as shown in Table 3 ▶. Further analysis is necessary to determine their functional relevance. In a few cases, analysis of these ion-binding sites has already yielded definitive functional insights (see §[Sec sec5]5).

### 6.2. PSI contribution to new Pfam families

One of the key goals of PSI has been to increase the structural coverage of protein family space. Pfam coverage by the current set of PSI structures now extends to 1630 families; for approximately 700 (∼43%) of these the PSI has provided the first structural representative. Over 150 of these Pfam families are populated by a single structure. Analysis of these first structural representatives representing 700 families indicates that over 175 of these structures contain some biologically relevant ligands. Of these, Zn^2+^ tops the list as the most frequently observed ligand in about 38 structures, followed by Mg^2+^ in 35 structures, Na^+^ in 23 structures, UNL in 16 structures and Ni^2+^ in 12 structures.

### 6.3. Biological relevance of common molecules bound to proteins

**Figure 5:**
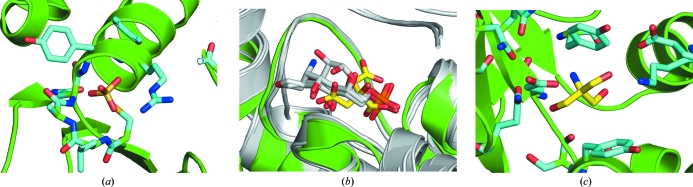
Common reagents bound in the active sites of proteins. The protein structures are shown in cartoon representation and colored green or gray. The bound ligands are drawn as sticks and are colored yellow (carbon), red (oxygen) and blue (nitrogen). The interacting residues are also drawn as sticks with their C atoms colored cyan. (*a*) An SO_4_^2−^ ion bound in the active site of protein YP_001181608.1 (PDB code 3gxg). (*b*) A citrate molecule bound to YP_001089791.1 (PDB code 3g68) helped to identify the potential active site and was supported by substrates (gray) bound to the same location in structurally similar proteins (gray; PDB codes 1mos, 2bpl, 2poc and 2v4m). (*c*) A Tris molecule bound in the active site of YP_001304206.1 (PDB code 3h3l).

Common reagents used during purification and crystallization, such as SO_4_^2−^, Cl^−^ or PO_4_^3−^ ions, buffer molecules such as Tris (2-­amino-2-hydroxymethyl-propane-1,3-diol) or citrate, and precipitants such as polyethylene glycols *etc*., often bind to proteins and are identified during structure refinement. In some cases, these bound reagents improve our understanding of putative binding sites on proteins and help to identify functionally relevant interactions by mimicking substrates. Here, we discuss three such examples (Fig. 5 ▶). A SO_4_^2−^ ion bound in the active site of YP_001181608.1 (PDB code 3gxg; http://www.topsan.org/Proteins/JCSG/3gxg) mimics a substrate phosphate moiety and lends support to its annotation as a phosphatase. Similarly, a citrate molecule helped to identify the active site in YP_001089791.1 (PDB code 3g68; http://www.topsan.org/Proteins/JCSG/3g68), where comparison of structurally similar proteins with a substrate bound in a similar location to the citrate led to the identification of likely active-site residues. In another example, the buffer molecule Tris is bound in the active site of the protein and emulates a sugar moiety in YP_001304206.1 (PDB code 3h3l; http://www.topsan.org/Proteins/JCSG/3h3l).

## 7. Data mining of ligands in crystal structures for improving methodology

In addition to being a rich source of functional clues, the ligands bound to PSI structures can also serve as a source of data to improve crystallographic methods and map interpretation. As an example, we examined the frequency with which various cryoprotectant reagents are observed in crystal structures. We limited our analysis to JCSG structures, since we also had the precise crystallization and cryoprotective conditions used for each structure. Analysis of about 800 structures indicates that the most frequently observed cryoprotectant is ethylene glycol (EDO), with a probability of ∼82% of being found in the structure if used in the crystallization/cryoprotective conditions, as shown in Table 4 ▶. The next on the list are polyethylene glycol 200 (PEG 200) and glycerol (GOL), with around a 56 or 55% chance, respectively, of being observed in the structure. A com­parative analysis performed with all of the structures in the PDB, although limited because the crystal growth and cryoprotective conditions are often missing from the deposition record, indicates a much smaller frequency of observation of these compounds in crystal structures. For example, of the 888 structures that list ethylene glycol as a crystallization/cryoprotective component in the PDB header, it is observed in only 184 (20.7%) of these structures. Similarly, only 723 (23.5%) structures indicate the presence of bound glycerol out of 3079 structures that report its use during crystallization. The high frequency of occurrence of these cryoprotectants in our structures suggests that more care should be taken in general to identify these molecules during model building and refinement if present in the crystallization/cryoprotective conditions and to include cryoprotectants in addition to the crystallization conditions in the PDB header.

## 8. Conclusions

We have provided an overview of the various types of ligands bound in PSI structures and have tabulated their relative frequencies. Furthermore, we have described how ligands are identified and modeled into the structures at JCSG. The sheer number and diversity of ligands found in JCSG structures, based on a rigorous and systematic interpretation of the electron-density maps, suggests that for many structures in the PDB, ligands may have been overlooked or not adequately characterized. The observation of bound ligands, including unknown ligands and common chemical reagents mimicking potential biological ligands, often enhances the functional annotation of novel, uncharacterized proteins and generates hypotheses which can be validated experimentally. The JCSG Ligand Search Server provides an easy tool to survey the large collection of novel PSI structures for their bound ligands.

## Figures and Tables

**Table 1:** Summary of ligands found in PSI and JCSG structures

Type	% observed in PSI structures	% observed in JCSG structures	No. of unique compounds/entities
Ligands	12.3	15.6	285
Peptides	1.2	0.6	
Cofactors	9.4	10.9	22
Metals	24.7	26.4	24
Non-metals	27.2	40.7	21
Organics	3.0	3.9	23
Buffers	10.7	19.3	14
Precipitants	5.2	14.2	14
Cryoprotectants	21.3	51.6	3
Overall	65.0	85.2	

**Table 2:** Unique ligands found in PSI structures

PDB code	Ligand name	Ligand ID	PSI center
1kph, 1kpi	Didecyldimethylammonium	10A	TBSGC
1z2l	Allantoate ion	1AL	NYSGXRC
1m33	3-Hydroxypropanoic acid	3OH	MCSG
1vr0	(2*R*)-3-Sulfolactic acid	3SL	JCSG
1y0g	2-[(2*E*,6*E*,10*E*,14*E*,18*E*,22*E*,26*E*)-3,7,11,15,19,23,27,31-Octamethyldotriaconta-2,6,10,14,18,22,26,30-octaenyl]phenol	8PP	NYSGXRC
1o8b	β-D-Arabinofuranose-5′-phosphate	ABF	MCSG
1tuf	Azelaic acid	AZ1	NYSGXRC
1y80	Co-5-methoxybenzimidazolylcobamide	B1M	SECSG
2b4b	*N*-Ethyl-*N*-[3-(propylamino)propyl]propane-1,3-diamine	B33	NYSGXRC
2a3l	Coformycin 5′-phosphate	CF5	CESG
2q09	3-[(4*S*)-2,5-Dioxoimidazolidin-4-yl]propanoic acid	DI6	NYSGXRC
2osu	6-Diazenyl-5-oxo-L-norleucine	DON	MCSG
2nw9	6-Fluoro-L-tryptophan	FT6	NESG
1p44	5-{[4-(9*H*-Fluoren-9-yl)piperazin-1-yl]carbonyl}-1*H*-indole	GEQ	TBSGC
2ou3	1*H*-Indole-3-carbaldehyde	I3A	JCSG
1x92	D-Glycero-D-mannopyranose-7-phosphate	M7P	MCSG
2gvc	1-Methyl-1,3-dihydro-2*H*-imidazole-2-thione	MMZ	NYSGXRC
1rtw	(4-Amino-2-methylpyrimidin-5-yl)methyl dihydrogen phosphate	MP5	NESG
2puz	*N*-(Iminomethyl)-L-glutamic acid	NIG	NYSGXRC
2od6	10-Oxohexadecanoic acid	OHA	JCSG
1n2h, 1n2i	Pantoyl adenylate	PAJ	TBSGC
1qpr	5-Phosphoribosyl-1-(β-methylene) pyrophos­phate	PPC	TBSGC
1xkl	2-Amino-4*H*-1,3-benzoxathiin-4-ol	STH	NESG
1bvr	*Trans*-2-hexadecenoyl-(*N*-acetyl-cysteamine)-thioester	THT	TBSGC
1lw4	3-Hydroxy-2-[(3-hydroxy-2-methyl-5-phosphono­oxymethylpyridin-4-ylmethyl)-amino]butyric acid	TLP	NYSGXRC

**Table 3:** Ligands bound to proteins of unknown function, excluding common crystallization reagents and cryoprotectants

Ligand†	Count	PDB codes
UNL	31	1vk91vpy2aam2g8l2i8d2opk2pnk2q9k2qdr2qe83cnx3d823e8o3ebt3ejv3ez03ezu3f7s3ff03fgv3fgy3fh13fka3flj3fsd3g163gi73giw3gzr3h3h3hrg
ZN	29	1q9u1sed1su01t8h1vk91vpy1xaf1xv21y7p1ylo2az42g7z2gnr2hek2i9w2oh32pg32pjs2r8c2rjb3chv3cjp3di43dza3e023e493feq3fm23h0n
NA	29	1nnh1nnw1q8c1sed1vk11vmf1vmh1vmj1yx11z671zl02asf2fbl2gkp2hhg2idl2il52okq2p0o2pnk2q3l2qsv2qzi2ra93dnx3f7c3frm3grd3h0n
MG	28	1tzz1z6n1zd01zke2a5z2f4i2fdr2g802gfq2h5n2hx02i3d2i712iec2nn52o352oy92p3p2p973bpd3c5p3cnx3cu33e2v3eo63etk3fa53hdg
CA	19	1sum1vly2arh2esh2g422gjv2i6h2pr72qng2rld3bdv3bfm3bvc3db73dt53en83fyb3g0k3h36
UNX	11	1xrg1xx71y811y821yb31ybx1yby1ybz1yd71yem1zd0
NI	10	1sum1xx72aj72o8q2ou62qe92qjv3bvc3d823h0n
FE	6	1sum2rg43bv63bww3dby3hc1
K	5	1vph1zl02aj72rgq3hc1
NO3	4	1t6a1t6s3dde3fov
MN	4	2p0n3ck23fij3gg7
COA	4	1q6y1y811yre2hqy
PT	2	1nnw1yem
PLM	2	1mgp1pzx
HG	2	1pvm1qz4
FMN	2	2i512iml
SNN	1	3esm
SIN	1	3cqy
SE	1	2arh
SAM	1	2qe6
SAH	1	3go4
RIP	1	1y7p
NDP	1	1xkq
NBZ	1	3bgu
NAP	1	1i36
NAD	1	2o2z
HXA	1	2g7z
GLC	1	2esr
GDP	1	2hek
CO3	1	3c9q
CO	1	2h9f
BR	1	2hek
BEZ	1	2q9r
AU	1	1she
ATP	1	3gbu

† The names of the ligands follow the IDs used in the PDB; their full names can be obtained from the Ligand Expo server (http://ligand-depot.rcsb.org/ld-search.html).

**Table 4:** Frequency of cryoprotectant reagents found as bound ligands in JCSG structures Values in parentheses correspond to occurances in all of the other structures in the PDB. These numbers are obtained from structures that report the use of these compounds in the crystal-growth conditions in their headers.

Cryo reagent†	No. of times used in crystallization/cryoprotective conditions	No. of times observed in structures	% observed
EDO	348 (888)	284 (184)	81.6 (20.7)
GOL	302 (3079)	167 (723)	55.3 (23.5)
MPD	106 (2558)	47 (373)	44.3 (14.6)
PEG 200	78	44	56.4
PEG 400	70	21	30.0

† Three-letter codes: EDO, ethylene glycol; GOL, glycerol; MPD, 2-methyl-2,4-pentanediol.
